# Maintenance interventions for overweight or obese children and adolescents who participated in a treatment program: study protocol for a systematic review

**DOI:** 10.1186/2046-4053-3-111

**Published:** 2014-10-03

**Authors:** Laila B van der Heijden, Edith JM Feskens, Arieke J Janse

**Affiliations:** 1 Department of Pediatrics, Hospital Gelderse Vallei, P.O. Box 9025, 6710 HN Ede, The Netherlands; 2 Division of Human Nutrition, Agrotechnology and Food Sciences Group, Wageningen University, P.O. Box 8129, 6700 EV Wageningen, The Netherlands

## Abstract

**Background:**

Childhood overweight and obesity are associated with significant health consequences. Early and successful treatment of this public health issue is necessary. Although several intervention programs for children result in weight loss or stabilisation in the short term, preventing relapse after weight loss remains an important challenge. Weight loss maintenance approaches in childhood are thought to be promising, but a structured overview of these maintenance interventions is lacking. The aim of the systematic review described in this protocol is to provide an overview of reports published about maintenance interventions in childhood overweight and obesity following initial treatment, in order to guide future directions in the development of maintenance programs for childhood obesity.

**Methods/design:**

The electronic databases PubMed, Embase, Cochrane Library, CINAHL, Web of Science, PsycINFO, Scopus, and SocINDEX will be searched for this review. Reference lists of eligible study reports will be scanned for relevant references. Article selection including risk of bias assessment will be performed independently in an unblinded standardised manner by three authors. All reports describing a maintenance intervention in overweight or obese children with a mean or median age of <18 years who have followed a treatment program, regardless of the type of intervention, will be included. Data extraction will be performed using a predesigned pilot-tested data extraction sheet that covers participant characteristics, details about the treatment preceding the maintenance intervention, and the maintenance intervention itself. Body mass index standard deviation score (BMI-SDS or BMI-Z-score) will be used to compare studies. If possible, a meta-analysis will be performed using the inverse-variance random-effects method. Studies that are not included in the meta-analysis will be described in a narrative way in tables and/or in the text.

**Discussion:**

This systematic review will give an overview of the existing knowledge on programs and initiatives aimed at long-term maintenance of a healthy or reduced weight in children and adolescents following initial treatment of overweight. It will form a basis for future research and practice in this area, a topic on which studies are scarce but highly necessary.

**Systematic review registration:**

PROSPERO CRD42014008698

## Background

Childhood overweight and obesity are associated with significant health consequences [1,2]. Besides cardiovascular morbidity and other medical conditions such as hepatic steatosis, cholelithiasis, orthopaedic complications, sleep apnoea, and polycystic ovary disease, obese children and adolescents and their families are at risk for psychological and social adjustment problems [3]. Early and successful treatment of this public health issue is necessary [4]. Although several intervention programs for children result in weight loss or stabilisation in the short term, preventing relapse after finishing a lifestyle intervention remains an important challenge. Many obese children regain weight after treatment, probably because weight loss/stabilisation techniques are abandoned and relapse occurs into inappropriate behaviours [5]. Maintenance programs that extend patient-therapist contacts beyond initial treatment have enhanced weight loss maintenance in adults [6]. Weight loss maintenance approaches in childhood are thought to be promising as well [7-10] and are highly requested in the fight against increasing weight in children. However, a structured overview of these maintenance interventions is lacking, and the overall effect of maintenance programs in childhood obesity has never been thoroughly evaluated. Furthermore, it is unknown which properties contribute to the success of a maintenance intervention. The aim of this systematic review is to fill in this gap of knowledge and to guide future directions in the development of maintenance programs.

### Objectives

The primary objective of this systematic review is to provide an overview of reports published about maintenance interventions in childhood overweight and obesity following initial treatment (review). In the remaining sections of this review protocol, the term overweight will be used to indicate both overweight and obesity.

The secondary objectives are the following:

– To consider the clinical effectiveness/efficacy of programs and initiatives aimed at long-term maintenance of a healthy or reduced weight in children and adolescents following initial treatment of overweight, using meta-analysis.

– To identify determinants within a maintenance intervention that contribute to the success of that intervention, both at study level using meta-regression (determinants such as type, intensity, and duration of the maintenance intervention), and at the individual level using descriptive statistics (determinants such as age and sex of the patient, parental support).

## Methods/design

### Criteria for considering studies for this review

#### *Types of studies*

Primarily, data from randomised controlled trials (RCTs) will be included in this systematic review. However, the value and appropriateness of these RCTs in assessing the efficacy of lifestyle interventions remains a contentious issue. Furthermore, a paucity of RCTs on maintenance interventions for childhood overweight is expected. Therefore, cohort studies (both prospective and retrospective, with and without control group) will also be included. Case reports will be excluded.

Given the expected clinical and methodological heterogeneity, there will be no minimum length of follow-up.

#### *Types of participants*

Study groups comprising children and adolescents aged less than 18 years at the commencement of a maintenance intervention will be included in this review. If a range with a maximum value of >18 years old is noted, the report will be included if the mean or median age is less than 18 years at the start of the maintenance intervention. All participants should have followed a treatment program for overweight, regardless of the type of intervention. In case the children are part of a family group receiving the intervention, the study will be included if data can be extracted separately for the children. Children with overweight due to a secondary cause or in the context of a syndrome will be excluded.

Non-responders are children with overweight or obesity who finished a treatment program but did not participate in the maintenance intervention or initiative. Drop-outs are defined as participants ending the maintenance program or initiative earlier than its normal termination. Both non-responders and drop-outs will be assessed in all studies included in the review.

Ideally (if this is published by the included reports), groups of patients undergoing a maintenance intervention will be compared with non-exposed control groups.

#### *Types of interventions*

All studies of maintenance or follow-up interventions or programs with the aim to maintain weight loss or a healthy lifestyle after a treatment program for overweight children will be included in this review. Components of the interventions can include diet and nutrition, exercise and physical activity, and lifestyle and social support. Studies are included if they teach skills specific to the maintenance of weight loss or if they otherwise try to stabilise or enhance adherence to a healthier lifestyle (e.g. with phone calls). There will be no restriction on the discipline(s) involved in the maintenance intervention and whether the intervention concerns one discipline or is multidisciplinary in nature. Furthermore, studies to be included will not be selected on the setting of the maintenance intervention (community health, primary or secondary or tertiary care).

#### *Types of outcome measures*

To meet the aforementioned primary and secondary objectives, the following primary and secondary outcomes are defined.

##### 

**Primary outcomes** The primary outcome measure for this review will be the body mass index standard deviation score (BMI-SDS or BMI-Z-score) based on measured height and weight. Studies with self-reported measurements of height and weight will not be included. BMI-SDS or BMI-Z-scores are preferred because these scores account for sex- and age-related changes over time. Second best parameters such as BMI and percentage overweight will be used when BMI-SDS or BMI-Z-scores cannot be extracted.

To be included, studies need to report a baseline and at least one post-intervention measurement.

##### 

**Secondary outcomes** There are no secondary outcomes.

### Search methods for identification of studies

#### *Electronic searches*

The following electronic databases will be searched: PubMed, Embase, Cochrane Library, CINAHL, Web of Science, PsycINFO, Scopus, and SocINDEX. Only studies published in English, Spanish, German, or Dutch will be included. There will be no publication date or publication status restrictions. Abstracts of conference presentations and posters will only be included if the results are not published otherwise. In that case, the investigators will be inquired about the study details.

The search strategy is added as Additional file 1. The search terms will be adapted for use in the different bibliographic databases.

#### *Searching other resources*

In addition to our electronic database search, reference lists of eligible study reports will be scanned for relevant references. Furthermore, subsequent trial registers will be checked: ClinicalTrials.gov, The European Union Clinical Trials Register, Current Controlled Trials, and the Netherlands Trial Register.

### Data collection and analysis

Search results will be merged using EndNote, and duplicate records of the same report will be removed.

#### *Selection of studies*

The retrieved records will be screened independently on title and selected for possible inclusion by LvdH, EF, and AJ. Obvious irrelevant reports will be excluded. Subsequently, abstracts of the remaining records will be assessed against predetermined inclusion criteria by the same three researchers. In case of rejection, it will be recorded why the study failed to meet the inclusion criteria.

For studies that appear to meet the inclusion criteria, or in cases when a definite decision cannot be made based on title and/or abstract alone, the full paper will be obtained for detailed assessment. This will be performed independently in an unblinded standardised manner by LvdH, EF, and AJ using a checklist on characteristics of the study and inclusion criteria. Furthermore, the full paper will be checked on internal and external validity (see paragraph on ‘Assessment of risk of bias in included studies’). The reason for exclusion of full-text articles will be provided and if any types of record are excluded, this will be stated. Disagreements will be discussed and, where possible, resolved by consensus after referring to the protocol. If necessary, a fourth person will be consulted. If needed, we will correspond with investigators to clarify study eligibility (it may be appropriate to request further information, such as missing results, at the same time). After final decisions on study inclusion, we will proceed to data collection.

#### *Data extraction and management*

A data extraction sheet will be developed and pilot-tested on ten randomly-selected included studies and refined accordingly. LvdH will extract the data from the included studies, and AJ will check the extracted data. Disagreements will be resolved by a discussion between LvdH and AJ. If no agreement is reached, it is planned that the third author (EF) decides. If information is not (or unclearly) reported, the authors will be contacted for further information, and the results of these contacts will be stated.

Information will be extracted from each included report on:

– Methods: study design, duration of study, length of follow-up.

– Characteristics of participants: total number of participants, setting, diagnostic criteria, age, sex, country, co-morbidity, socio-demographics, ethnicity, participant selection process (in- and exclusion criteria), non-responders.

– Type of treatment preceding the maintenance intervention.

– Type of maintenance intervention(s): total number of intervention/control groups. For each intervention/control group: number of participants, focus of intervention (parents, children, both), contents of intervention (components used), disciplines involved, length of intervention, number of sessions, duration/frequency/timing of delivery, participant responsiveness, drop-outs, concurrent interventions, unintended exposure.

– Outcome measures: abovementioned primary and secondary outcomes (if possible to extract), with definition, unit of measurement, time-point(s). For each outcome: sample size, missings, summary of data, estimate of effect, subgroup analysis (if applicable).

A preliminary literature survey showed substantial differences in 1) the treatment preceding the maintenance interventions, 2) the maintenance interventions themselves, and 3) the outcome measures (clinical heterogeneity). If the data extraction process will reveal descriptive factors or outcome parameters that are potentially useful but not yet included in the initial data extraction form, the data extraction form will be revised.

#### *Assessment of risk of bias in included studies*

Assessing the risk of bias in the included studies will be performed according to the Risk of Bias Assessment Tool form attached as Additional file 2. This form is based on the ‘risk of bias’ tool developed by The Cochrane Collaboration to assess risk of bias in randomised controlled trials [11], supplemented with items extracted from the Newcastle-Ottawa quality assessment tool [12] and from the Agency for Healthcare Research and Quality (AHRQ) publication of Viswanathan et al. [13]. The tool added as Additional file 2 consists of items that cover five domains of bias (selection, performance, attrition, detection, and reporting), as well as an ‘other bias’ category to capture other potential threats to validity. Three review authors will assess the risk of bias for each study. Review authors will not be blinded with respect to study authors, institution, or journal as they are familiar with the literature. Any disagreements will be resolved by a discussion. Results of the risk of bias assessment will be presented in the final report. The findings will be taken into consideration when analysing the data and drawing conclusions, using sensitivity analysis where appropriate.

Assessment of the risk of selection bias in RCTs will include the criteria ‘random sequence generation’ and ‘allocation concealment’ (‘low risk’, ‘high risk’ or ‘unclear risk’ of bias). When the study investigators clearly describe a random component in the sequence generation process, a judgement of ‘low risk’ will be assigned to sequence generation. Allocation concealment will be assessed as ‘low risk’ if the method used to prevent the participants or investigators enrolling participants to foresee the group assignment is described. All non-randomised studies will be assessed as ‘high risk’ for both sequence generation and allocation concealment. For non-randomised trials and observational studies, detailed criteria on selection bias (concerning comparability of groups, confounding, and adjustment) are included. Furthermore, in cohort studies, the representativeness of the exposed cohort will be evaluated using the presented inclusion and exclusion criteria and selection procedure. Because of the expected clinical heterogeneity of the maintenance cohorts, the cohorts presented in the included reports will be accurately described in our paper.

The item ‘blinding’ will be used to assess the risk of performance and detection bias. A judgement of ‘low risk’ of bias will be assigned when the outcome assessors are blinded for the allocated intervention. With respect to performance and detection bias, in cohort studies, also the comparability of cohorts will be checked, as well as the impact from concurrent interventions or an unintended exposure that might bias the results.

With respect to attrition bias (completeness of sample, follow-up, and data), ‘low risk’ will be assigned to a study when the proportion of missing outcome data is well-described and relatively balanced between the intervention groups, and the reasons for incomplete outcome data across intervention groups are provided and unlikely to be related to the true outcome.

The risk of reporting bias will be considered low if a study protocol is available, and all of the study’s prespecified (primary and secondary) outcomes that are of interest have been reported in the prespecified way.

If studies will be excluded from the review or any subsequent analyses on the basis of the risk of bias, this will be described and the reasons for the exclusions will be explained.

A preliminary literature survey on the topic of this review revealed a wide variety of study designs (methodological heterogeneity). In the Risk of Bias Assessment Tool added as Additional file 2, important criteria to take into account when encountering non-randomised trials or observational studies are enumerated, as well as items to consider in the assessment of RCTs. If other study designs will emerge during the study selection process, additional criteria will be added to the risk of bias assessment where appropriate to warrant an appropriate risk of bias assessment for each study type. At any time this will be described in the final paper.

#### *Measures of treatment effect, data synthesis, and investigation of heterogeneity*

BMI-SDS or BMI-Z-scores will be collected to compare studies in the results section of this review. When BMI-SDS or BMI-Z-scores are not reported, these will be calculated or extracted from the data given in the report. If calculation is not possible, the authors will be contacted. If BMI-SDS or BMI-Z-scores are not available anyhow, the absolute change in BMI or percentage overweight will be used as second best outcome variable.

To decide as to whether a meta-analysis is appropriate, the amount of data available as well as the clinical (variability in terms of participants, interventions, and outcomes) and methodological (variability in study design and risk of bias) diversity will be taken into account. The results of studies using the same type of intervention and comparator will be pooled using a meta-analysis on BMI-SDS or BMI-Z-score. The means will be used to determine the difference between means of the BMI-SDS or BMI-Z-score (change in BMI-SDS or BMI-Z-score during the maintenance intervention). Corresponding measures of precision (standard deviations, standard errors, or 95% confidence intervals) of the means and the difference between means will be extracted. If not reported, the SD will be derived from the reported standard error (SE) of the mean or can be obtained from confidence intervals, *t* statistics, and *P* values. If SDs are given rather than SEs, the SE will be calculated by dividing the SD by the square root of *n*. If the SD or SE of the mean change is lacking, the following formula will be used: standard deviation_difference_ = square root ([variance_baseline_ + variance_follow-up_] - [2 × correlation_baseline, follow-up_ × standard deviation_baseline_ × standard deviation_follow-up_]) [5,14]. The median correlation between the baseline and post-intervention BMI-SDS will be calculated from the selected studies, compared with other reports, and used in this formula.

The *I*^2^ statistic will be used to assess whether the observed variability in study results is greater than expected to occur by chance (statistical heterogeneity). Thresholds for the interpretation of *I*^2^ will be according to the guidance for Cochrane reviews [11]:

– 0%–40%: might not be important.

– 30%–60%: may represent moderate heterogeneity*.

– 50%–90%: may represent substantial heterogeneity*.

– 75%–100%: considerable heterogeneity*.

*The importance of the observed value of *I*^2^ will depend on the magnitude and direction of the effects and the strength of evidence for heterogeneity.

If there is substantial or considerable heterogeneity in results, and particularly, if there is inconsistency in the direction of effect, it may be misleading to quote an average value for the intervention effect. The consistency of results across the studies will influence the decision whether or not to combine results in a meta-analysis.

Assuming that there is no common treatment effect for all included studies, the inverse-variance random-effects meta-analysis method will be used. Where a study reports data immediately post-intervention and at a subsequent follow-up time point, only the data immediately post-intervention will be included in the meta-analysis. Analysis will be conducted using the statistical program R version 2.15.1 with the metafor package. Data will be presented in forest plots.

Studies that are not included in the meta-analysis will be described in a narrative way in tables and/or in the text. If appropriate, these studies will be organised into groupings or clusters (e.g. by intervention type, population group, or setting).

#### *Unit of analysis issues*

If studies with multiple treatment groups are present, all intervention groups that meet the criteria for including studies in this review (if investigated alone) will be included and compared with the control arm (if applicable). If needed, groups will be combined to create a single pair-wise comparison.

If cluster-randomised trials are present, this will be noted in the final report and it will be explicitly stated how data were handled: either by conducting the analysis at the same level as the allocation, or by statistical methods that allow analysis at the level of the individual while accounting for the clustering in the data.

#### *Dealing with missing data*

Whenever possible, original investigators will be contacted to request missing data. Assumptions of any methods used to cope with the missing data will be made explicit. If appropriate, sensitivity analyses will be performed to assess how sensitive results are to reasonable changes in the assumptions that are made. Furthermore, the potential impact of the missing data on the findings of the review will be discussed in the Discussion section.

#### *Assessment of reporting biases*

If possible, for each trial, the effect by the inverse of its standard error will be plotted. The symmetry in the funnel plots will be assessed both visually, [15] and formally with Egger’s test. Asymmetry can result not only from non-publication of small studies with negative results but also from selective outcome reporting or poor methodological quality leading to inflated effects. The results and interpretation of the assessment of reporting biases will be discussed in the Discussion section.

#### *Subgroup analysis*

Subgroup analyses will be performed if enough data is available in order to explore possible heterogeneity (taking into account that investigations of heterogeneity when there are very few studies are of questionable value) and to investigate the effectiveness of maintenance interventions for particular patient groups or using different types of intervention. As the age of patients, the different components used in the maintenance programs, and the intensity of the maintenance programs are expected to mainly influence the effect of the maintenance programs in overweight children, subgroup analyses will be performed with subgroups defined according to these determinants.

If more than ten studies are available in the meta-analysis, meta-regression will be considered to investigate how different characteristics (for example intensity and duration of maintenance contact) are associated with the intervention effect; in other words: to identify aspects within a maintenance program that contribute to the success of that program.

All analyses will be performed using the statistical program R version 2.15.1 with the metafor package.

#### *Sensitivity analysis*

If appropriate, sensitivity analyses will be performed to explore the degree to which the main findings of the review are affected by changes in the methods or in the data used from individual studies. Issues suitable for sensitivity analysis will be identified during the review process.

## Discussion

Early and successful treatment of childhood overweight is of the utmost importance as it is known to have significant impact on both physical and psychosocial health [1,2]. Currently, two great challenges of lifestyle interventions are the maintenance of achieved weight loss/stabilisation and the prevention of relapse into inappropriate health behaviours.

This systematic review will give an overview of the existing knowledge on programs and initiatives aimed at long-term maintenance of a healthy or reduced weight in children and adolescents following initial treatment of overweight. It will form a basis for future research and practice in this area, a topic on which studies are scarce but highly necessary.

## Abbreviations

BMI: body mass index; BMI-SDS: body mass index standard deviation score; RCT: randomised controlled trial.

## Competing interests

The authors declare that they have no competing interests.

## Authors’ contributions

LvdH designed the protocol and wrote the manuscript. EF and AJ participated in the design of the study and helped to draft the manuscript. All authors read and approved the final manuscript.

## Supplementary Material

Additional file 1 **Search strategy.** Detailed description of search strategy using the following electronic databases: PubMed, Embase, Cochrane Library, CINAHL, Web of Science, PsycINFO, Scopus, and SocINDEX. Click here for file

Additional file 2 **Risk of Bias Assessment Tool.** Risk of Bias Assessment Tool form that will be used to assess the risk of bias in included studies in this systematic review. This tool is based on the ‘risk of bias’ tool developed by The Cochrane Collaboration to assess risk of bias in randomised controlled trials [11], supplemented with items extracted from the Newcastle-Ottawa quality assessment tool [12] and from the Agency for Healthcare Research and Quality (AHRQ) publication of Viswanathan et al. [13]. Click here for file
