# Supplementary material for: Maintenance interventions for overweight or obese children and adolescents who participated in a treatment program: study protocol for a systematic review
Source: Syst Rev. 2014 Oct 3;3:111. doi: 10.1186/2046-4053-3-111 (PMC4196205; doi:10.1186/2046-4053-3-111)
Supplement: Additional file 1 — Search strategy. Detailed description of search strategy using the following electronic databases: PubMed, Embase, Cochrane Library, CINAHL, Web of Science, PsycINFO, Scopus, and SocINDEX. [file 2046-4053-3-111-S1.doc]

**Additional file 1: Search strategy**

Pubmed

Filters activated: Dutch, English, Spanish, French, German.

1. "Overweight"[Mesh] OR "Obesity"[Mesh:noexp] OR "Obesity, Morbid"[Mesh] OR "Obesity, Abdominal"[Mesh] OR "Adiposity"[Mesh:noexp] OR overweight[Title/Abstract] OR obesity[Title/Abstract] OR obese[Title/Abstract] OR obesitas[Title/Abstract] OR adiposity[Title/Abstract] OR adipositas[Title/Abstract] OR excessive weight[Title/Abstract]
2. child[Title/Abstract] OR children[Title/Abstract] OR childhood[Title/Abstract] OR adolescent[Title/Abstract] OR adolescents[Title/Abstract] OR teen[Title/Abstract] OR teens[Title/Abstract] OR teenager[Title/Abstract] OR teenagers[Title/Abstract] OR youth[Title/Abstract] OR youths[Title/Abstract] OR adolescence[Title/Abstract] OR youngster[Title/Abstract] OR youngsters[Title/Abstract] OR schoolchildren[Title/Abstract] OR pediatric[Title/Abstract] OR pediatrics[Title/Abstract] OR paediatric[Title/Abstract] OR paediatrics[Title/Abstract] OR girl[Title/Abstract] OR girls[Title/Abstract] OR boy[Title/Abstract] OR boys[Title/Abstract] OR "Child"[Mesh] OR "Adolescent"[Mesh] OR "Minors"[Mesh]
3. #1 AND #2
4. therapy[Title/Abstract] OR therapies[Title/Abstract] OR treatment[Title/Abstract] OR treatments[Title/Abstract] OR intervention[Title/Abstract] OR interventions[Title/Abstract] OR program[Title/Abstract] OR programs[Title/Abstract] OR programme[Title/Abstract] OR programmes[Title/Abstract] OR strategy[Title/Abstract] OR strategies[Title/Abstract] OR care[Title/Abstract] OR approach[Title/Abstract] OR approaches[Title/Abstract] OR "therapy"[Subheading]
5. maintenance[Title/Abstract] OR follow-up[Title/Abstract] OR followup[Title/Abstract]
6. #4 AND #5
7. post-treatment[Title/Abstract] OR posttreatment[Title/Abstract] OR aftercare[Title/Abstract]
8. #6 OR #7
9. #3 AND #8
10. #9 NOT "Animals"[Mesh]) NOT ("Animals"[Mesh]) AND "Humans"[Mesh])

The Cochrane Library (Wiley)

1. MeSH descriptor: [Overweight] explode all trees
2. MeSH descriptor: [Obesity] this term only
3. MeSH descriptor: [Obesity, Abdominal] explode all trees
4. MeSH descriptor: [Obesity, Morbid] explode all trees
5. MeSH descriptor: [Adiposity] this term only
6. MeSH descriptor: [Child] explode all trees
7. MeSH descriptor: [Adolescent] explode all trees
8. MeSH descriptor: [Minors] explode all trees
9. overweight or obesity or obese or obesitas or adiposity or adipositas or excessive weight:ti,ab,kw
10. #9 or #1 or #2 or #3 or #4 or #5
11. child or childhood or adolescent or teen or teenager or youth or adolescence or youngster or schoolchildren or pediatric or pediatrics or girl or boy:ti,ab,kw
12. #11 or #6 or #7 or #8
13. therapy or treatment or intervention or program or strategy or care or approach:ti,ab,kw
14. maintenance or follow-up or followup:ti,ab,kw
15. #13 and #14
16. post-treatment or posttreatment or aftercare:ti,ab,kw
17. #15 or #16
18. #10 and #13
19. #17 and #18

CINAHL, PsycINFO, SocINDEX (EBSCO)

1. TI overweight OR AB overweight OR TI obesity OR AB obesity OR TI obese OR AB obese OR TI obesitas OR AB obesitas OR TI adiposity OR AB adiposity OR TI adipositas OR AB adipositas
2. TI excessive weight OR AB excessive weight
3. S1 OR S2
4. TI child OR AB child OR TI children OR AB children OR TI childhood OR AB childhood OR TI adolescent OR AB adolescent OR TI adolescents OR AB adolescents OR TI teen OR AB teen
5. TI teens OR AB teens OR TI teenager OR AB teenager OR TI teenagers OR AB teenagers OR TI youth OR AB youth OR TI youths OR AB youths OR TI adolescence OR AB adolescence
6. TI youngster OR AB youngster OR TI youngsters OR AB youngsters OR TI schoolchildren OR AB schoolchildren OR TI pediatric OR AB pediatric OR TI pediatrics OR AB pediatrics TI paediatric OR AB paediatric
7. TI paediatrics OR AB paediatrics OR TI girl OR AB girl OR TI girls OR AB girls OR TI boy OR AB boy OR TI boys OR AB boys
8. S4 OR S5 OR S6 OR S7
9. TI therapy OR AB therapy OR TI therapies OR AB therapies OR TI treatment OR AB treatment OR TI treatments OR AB treatments OR TI intervention OR AB intervention OR TI interventions OR AB interventions
10. TI program OR AB program OR TI programs OR AB programs OR TI programme OR AB programme OR TI programmes OR AB programmes OR TI strategy OR AB strategy OR TI strategies OR AB strategies
11. TI care OR AB care OR TI approach OR AB approach OR TI approaches OR AB approaches
12. TI maintenance OR AB maintenance OR TI follow up OR AB follow up OR TI followup OR AB followup
13. S9 OR S10 OR S11
14. S12 AND S13
15. TI post-treatment OR AB post-treatment OR TI posttreatment OR AB posttreatment OR TI aftercare OR AB aftercare
16. S14 OR S15
17. S3 AND S8
18. S16 AND S17
19. S18 Limiters – Human; Language: Dutch/Flemish, English, French, German, Spanish

EMBASE

1. ‘overweight’/exp OR ‘obesity’/exp OR ‘adiposity’/exp
2. overweight:ab,ti OR obesity:ab,ti OR obese:ab,ti OR obesitas:ab,ti OR adiposity:ab,ti OR adipositas:ab,ti OR ‘excessive weight’:ab,ti
3. #1 OR #2
4. ‘child’/exp OR ‘adolescent’/exp OR ‘minors’/exp
5. child:ab,ti OR children:ab,ti OR childhood:ab,ti OR adolescent:ab,ti OR adolescents:ab,ti OR teen:ab,ti OR teens:ab,ti OR teenager:ab,ti OR teenagers:ab,ti OR youth:ab,ti OR youths:ab,ti OR adolescence:ab,ti OR youngster:ab,ti OR youngsters:ab,ti OR schoolchildren:ab,ti OR pediatric:ab,ti OR pediatrics:ab,ti OR paediatric:ab,ti OR paediatrics:ab,ti OR girl:ab,ti OR girls:ab,ti OR boy:ab,ti OR boys:ab,ti
6. #4 OR #5
7. #3 AND #6
8. therapy:ab,ti OR therapies:ab,ti OR treatment:ab,ti OR treatments:ab,ti OR intervention:ab,ti OR interventions:ab,ti OR program:ab,ti OR programs:ab,ti OR programme:ab,ti OR programmes:ab,ti OR strategy:ab,ti OR strategies:ab,ti OR care:ab,ti OR approach:ab,ti OR approaches:ab,ti
9. maintenance:ab,ti OR ‘follow up’:ab,ti OR followup:ab,ti
10. #8 AND #9
11. ‘post treatment’:ab,ti OR posttreatment:ab,ti OR aftercare:ab,ti
12. #10 OR #11
13. #7 AND #12
14. #13 AND ([dutch]/lim OR [english]/lim OR [french]/lim OR [german]/lim OR [spanish]/lim) AND [humans]/lim

Web of Science

1. TOPIC: (overweight) OR TOPIC: (obesity) OR TOPIC: (obese) OR TOPIC: (obesitas) OR TOPIC: (adiposity) OR TOPIC: (adipositas OR TOPIC: (‘excessive weight)
2. TOPIC: (child) OR TOPIC: (children) OR TOPIC: (childhood) OR TOPIC: (adolescent) OR TOPIC: (adolescents) OR TOPIC: (teen) OR TOPIC: (teens) OR TOPIC: (teenager) OR TOPIC: (teenagers) OR TOPIC: (youth) OR TOPIC: (youths) OR TOPIC: (adolescence) OR TOPIC: (youngster) OR TOPIC: (youngsters) OR TOPIC: (schoolchildren) OR TOPIC: (pediatric) OR TOPIC: (pediatrics) OR TOPIC: (paediatric) OR TOPIC: (paediatrics) OR TOPIC: (girl) OR TOPIC: (girls) OR TOPIC: (boy) OR TOPIC: (boys)
3. #1 AND #2
4. TOPIC: (therapy) OR TOPIC: (therapies) OR TOPIC: (treatment) OR TOPIC: (treatments) OR TOPIC: (intervention) OR TOPIC: (interventions) OR TOPIC: (program) OR TOPIC: (programs) OR TOPIC: (programme) OR TOPIC: (programmes) OR TOPIC: (strategy) OR TOPIC: (strategies) OR TOPIC: (care) OR TOPIC: (approach) OR TOPIC: (approaches)
5. TOPIC: (maintenance) OR TOPIC: (‘ follow up’) OR TOPIC: (followup)
6. TOPIC: (post-treatment) OR TOPIC: (posttreatment) OR TOPIC: (aftercare)
7. #4 AND #5
8. #6 OR #7
9. #3 AND #8
10. #9 Refined by: LANGUAGES=( ENGLISH OR GERMAN OR FRENCH OR SPANISH OR DUTCH )

Scopus

1. TITLE-ABS-KEY(overweight) OR TITLE-ABS-KEY(obesity) OR TITLE-ABS-KEY(obese) OR TITLE-ABS-KEY(obesitas) OR TITLE-ABS-KEY(adiposity) OR TITLE-ABS-KEY(adipositas) OR TITLE-ABS-KEY('excessive weight')
2. TITLE-ABS-KEY(child) OR TITLE-ABS-KEY(children) OR TITLE-ABS-KEY(childhood) OR TITLE-ABS-KEY(adolescent) OR TITLE-ABS-KEY(adolescents) OR TITLE-ABS-KEY(teen) OR TITLE-ABS-KEY(teens) OR TITLE-ABS-KEY(teenager) OR TITLE-ABS-KEY(teenagers) OR TITLE-ABS-KEY(youth) OR TITLE-ABS-KEY(youths) OR TITLE-ABS-KEY(adolescence) OR TITLE-ABS-KEY(youngster) OR TITLE-ABS-KEY(youngsters) OR TITLE-ABS-KEY(schoolchildren) OR TITLE-ABS-KEY(pediatric) OR TITLE-ABS-KEY(pediatrics) OR TITLE-ABS-KEY(paediatric) OR TITLE-ABS-KEY(paediatrics) OR TITLE-ABS-KEY(girl) OR TITLE-ABS-KEY(girls) OR TITLE-ABS-KEY(boy) OR TITLE-ABS-KEY(boys)
3. #1 AND #2
4. TITLE-ABS-KEY(therapy) OR TITLE-ABS-KEY(therapies) OR TITLE-ABS-KEY(treatment) OR TITLE-ABS-KEY(treatments) OR TITLE-ABS-KEY(intervention) OR TITLE-ABS-KEY(interventions) OR TITLE-ABS-KEY(program) OR TITLE-ABS-KEY(programs) OR TITLE-ABS-KEY(programme) OR TITLE-ABS-KEY(programmes) OR TITLE-ABS-KEY(strategy) OR TITLE-ABS-KEY(strategies) OR TITLE-ABS-KEY(care) OR TITLE-ABS-KEY(approach) OR TITLE-ABS-KEY(approaches)
5. TITLE-ABS-KEY(maintenance) OR TITLE-ABS-KEY('follow up') OR TITLE-ABS-KEY(followup)
6. #4 AND #5
7. TITLE-ABS-KEY(post-treatment) OR TITLE-ABS-KEY(posttreatment) OR TITLE-ABS-KEY(aftercare)
8. #6 OR #7
9. #3 AND #8
